# Adopting the COM‐B model and TDF framework in oral and dental research: A narrative review

**DOI:** 10.1111/cdoe.12677

**Published:** 2021-07-16

**Authors:** Heather Buchanan, Jonathon Timothy Newton, Sarah R Baker, Koula Asimakopoulou

**Affiliations:** ^1^ Division of Rehabilitation, Ageing & Wellbeing Medical School University of Nottingham Nottingham UK; ^2^ Faculty of Dentistry, Oral and Craniofacial Sciences King’ College London Guy’s Hospital London UK; ^3^ Oral Health Dentistry and Society School of Clinical Dentistry University of Sheffield Sheffield UK

**Keywords:** Behavioral science, Psychosocial aspects of oral health

## Abstract

**Background:**

Recent advances in the psychological understanding of health‐related behaviour have focused on producing a comprehensive framework to model such behaviour. The Capability‐Opportunity‐Motivation‐Behaviour (COM‐B) and its associated Theoretical Domains Framework (TDF) allow researchers to classify psychological and behavioural constructs in a consistent and transferable manner across studies.

**Aim:**

To identify oral and dental health‐related studies that have used the TDF and/or COM‐B as frameworks to guide research and examine the ways in which these concepts have been practically used in such research.

**Method:**

Narrative review of published literature. To be included, the paper had to (1) state that the TDF or COM‐B had been used and to have targeted at least one construct identified in either framework, (2) include primary empirical data, (3) focus on a behaviour directly related to oral or dental‐related health (eg brushing, applying fluoride varnish, flossing) and/or attitudes, intentions and beliefs related to the behaviour. Studies could include any research design, and participants of any age or gender and include patients, parents or dental health professionals.

**Findings:**

Nine studies were identified that had drawn on the COM‐B and/or TDF as the framework for their research. Seven of the studies were based on the TDF only, with one employing both the COM‐B and Health Belief Model, and one using the TDF with COM‐B. The nine studies covered a broad range of oral health‐related behaviours including child tooth brushing, fluoride varnish application and non‐ or micro‐invasive management of proximal caries lesions. The populations in the studies included dentists, dental teams and parents of children. All studies adopted only a subset of the constructs within the TDF, often without justification.

**Conclusions:**

It is encouraging that oral health researchers are adopting standardized psychological frameworks to develop their research and oral health interventions. Future work should build on the small number of studies identified in this review and consider using standardized tools to do so.

## 1 BACKGROUND

Including theory in the design of behaviour change strategies is essential, as interventions based on the theoretical modelling of behaviour have been shown to be more effective than non‐theory‐based interventions.^1^, ^2^, ^3^ However, one issue that has faced researchers seeking to develop theory‐based interventions for oral health‐related behaviour—as well as in the wider health field—was the choice of an appropriate theoretical framework given the large number of frameworks (often with overlapping constructs) available in the psychological literature.

**FIGURE 1:**
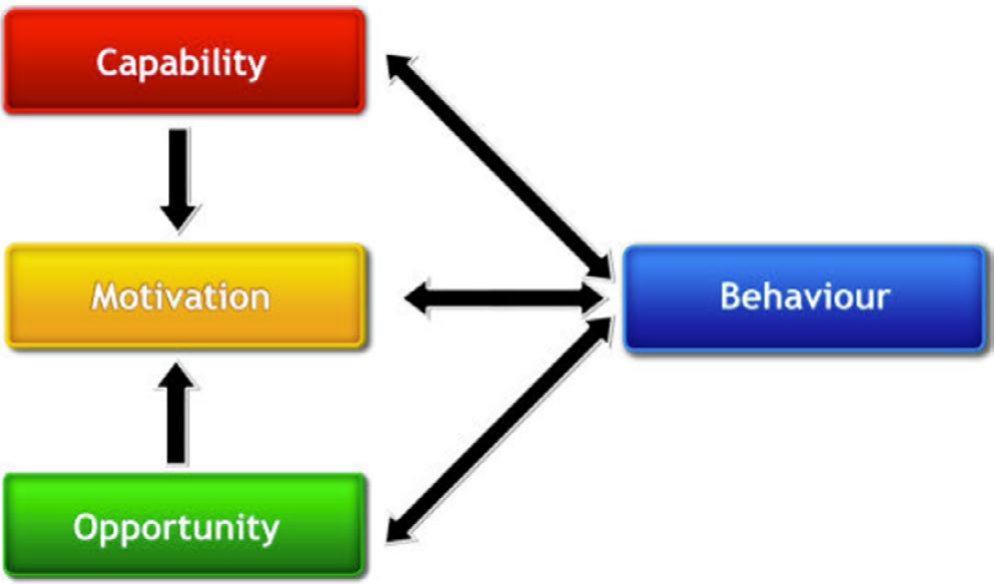
The COM‐B model ^11^

Over the last decade, there has been a concerted effort to synthesize the common elements of different models into a single framework for understanding the psychological determinants of behaviour and to inform the design of interventions.^4^ The result has been the development of the Capability‐Opportunity‐Motivation‐Behaviour Model (COM‐B; Figure 1), and the Theoretical Domains Framework (TDF; Table 1).

**TABLE 1:** The Theoretical Domains Framework ^8^

TDF Domain	Definition
Knowledg	Information relating to the behaviour
Skills	An ability or proficiency acquired through practice
Social/Professional Role and Identit	A coherent set of behaviours and displayed personal qualities of an individual in a social or work setting
Beliefs about Capabilities	Acceptance of the truth, reality or validity about an ability, talent or facility
Optimism	The confidence that things will happen for the best or that desired goals will be attained
Beliefs about Consequences	Acceptance of the truth, reality or validity about outcomes of a behaviour in a given situation
Reinforcement	Increasing the probability of a response by arranging a dependent relationship, or contingency, between the response and a given stimulus
Intentions	A conscious decision to perform a behaviour or a resolve to act in a certain way
Goals	Mental representations of outcomes or end states that an individual wants to achieve
Memory, Attention and Decision Processes	The ability to retain information, focus selectively on aspects of the environment and choose between two or more alternatives
Environmental Context and Resources	Any circumstance of a person's situation or environment that discourages or encourages the development of skills and abilities, independence, social competence and adaptive behaviour
Social influences	Those interpersonal processes that can cause individuals to change their thoughts, feelings or behaviours
Emotion	A complex reaction pattern, involving experiential, behavioural, and physiological elements, by which the individual attempts to deal with a personally significant matter or event
Behavioural Regulation	Anything aimed at managing or changing objectively observed or measured actions

According to the COM‐B Model (see Figure 1), an individual's behaviour is the result of an interaction between the individual's *Capability* to perform the behaviour, the *Opportunity*to engage in the behaviour and *Motivation* which directs the occurrence of the behaviour at a given moment.^4^, ^5^ *Capability* has both psychological and physical components, the psychological component includes knowledge of the behaviour and the ability to comprehend information and to reason, while the physical component of Capability includes skill, dexterity and strength required for the behaviour. Likewise, *Opportunity* has two subcomponents as follows: Physical opportunity (created by environment, for example access to resources) and social (norms and expectations of behaviour). *Motivation* can either be automatic (such as habits) or reflective (motivational elements such as planning and decision‐making).^5^ It has been proposed that for behaviour change to take place within oral health settings all three components need to be carefully considered.^6^

The Theoretical Domains Framework (TDF) consists of 14 constructs which are mapped onto the COM‐B model in order to further analyse the proximal determinants of behaviours.^7^ It was produced using a consensus methodology combining 83 behaviour change theories which together contained 128 psychological constructs.^8^ Table 1 lists the TDF domains together with a brief definition for each term, while Table 2 identifies the mapping of the TDF domains to the COM‐B model.

**TABLE 2:** The COM‐B Model and its relation to the TDF

COM‐B component		TDF domain
Capability	Psychological	Knowledge Skills Memory, Attention and Decision Processes
	Physical	Behavioural regulation Skills
Opportunity	Social	Social influences
	Physical	Environmental context & resources
Motivation	Reflective	Social/Professional Role & Identity Beliefs about capability Optimism Beliefs about consequences Intentions Goals
	Automatic	Social/Professional Role & Identity Optimism Reinforcement Emotion

Note

Adapted from Cane et al (2012) ^8^

The aim of the TDF was to help provide an assessment of the broad behavioural barriers and enablers that are thought to underpin behaviour change and, as such, inform the design of appropriately targeted interventions. In relation to the wider health field outside of oral/dental health, citations of the TDF and the COM‐B have increased exponentially since their original development.^9^ In their recent review of health behaviour interventions, Codwell and Dyson found that the frameworks had been used to design interventions for a wide range of populations including children and young people, parents, overweight pregnant women, pregnant smokers, smokers, sedentary office workers, overweight people, heterosexual men and people with hypertension. Most of these interventions targeted diet and exercise as the behavioural outcome of interest.

The great strength of COM‐B and the TDF is the ability to use a common taxonomic framework to synthesize interventions across a range of different settings and research designs, whereas a potential weakness is an opaqueness and lack of clarity pertaining to *how* constructs within them should be used in practice.^10^, ^11^ Nevertheless, oral health researchers have started engaging with both frameworks in order to ground their behavioural intervention work into concrete behavioural science theory. Currently, it is not known to what extent (or how) these frameworks have been used in practice, what questions they have tended to address, with what population groups or which, if any, of their components have been shown to be helpful across oral health interventions. Better understanding of how COM‐B and the TDF can be used to shape oral and dental health research is paramount if these frameworks are going to be used routinely in this field.

The aim of the review was to identify oral and dental research studies that have used the TDF and/or COM‐B as frameworks to guide the conduct of research, that is, the study's choice of study design, constructs, measures, analytic strategy and to understand how they have done so. It is envisaged that in this way the common elements across studies can be identified and implications of using the COM‐B and TDF to enhance oral health‐related behaviour studies can be outlined.

Narrative reviews are often seen as ‘state of the science’ type reviews and are useful for providing a narrative synthesis of previously published information. They are also beneficial for providing a broad perspective particularly on the history or development of a topic.^12^ For these reasons we chose to carry out a narrative review the results of which appear next.

## 2 METHOD

We searched for papers that had used either COM‐B or TDF to inform the study design. To be included in the review, the paper had to state clearly that the TDF or COM‐B had been used and to have targeted at least one construct identified in the COM‐B or TDF. Judgements were made on the basis of information in the paper regarding the use of one/both frameworks.

We included studies of any design which analysed primary empirical data including randomized controlled trials, controlled clinical trials, cohort studies, case‐control studies, qualitative interview/focus group studies and those using mixed methods.

Studies could include participants of any age or gender and could include patients, parents and dental health professionals. We included studies focusing on a behaviour directly related to oral or dental‐related health (brushing, applying fluoride varnish, flossing) and/or attitudes, intentions and beliefs related to the behaviour.

As the aim of the review was to identify oral and dental health‐related studies, we only included health behaviours (eg smoking, alcohol consumption) that have been linked to certain oral health conditions (eg periodontal disease, oral cancer) if they were specifically situated within / targeted to the oral/dental healthcare context (eg providing smoking cessation advice in the dental clinic). Instead of using specific search terms, we located studies for inclusion in the review, by searching SCOPUS for papers which had citations of either of two key behaviour change papers that originally proposed and described COM‐B and TDF. These were as follows:(Cane et al.,2012)^8^ and Michie et al.,(2011)^13^

For the purpose of this review, we did not search the grey literature as we were specifically interested in studies that had been peer reviewed and published. There were no language or date restrictions for papers.

Three authors (JTN, KA and HB) conducted the searches in August 2019. Two reviewers (KA and HB) completed the screening of papers for inclusion and extraction of data. JTN was designated as a third reviewer. The two reviewers assessed the studies by examining titles, keywords and abstracts. Any papers that did not meet the inclusion criteria were rejected at this stage. There was a disagreement on one study, and the two reviewers were able to resolve this through discussion. Full papers were retrieved for all studies that appeared to meet the inclusion criteria. Further review led to the rejection of some papers at this stage.

The two reviewers independently extracted data for each study on a data sheet designed for the study. We based the structure of our extraction sheet on consultation with experts on the development of the COM‐B and TDF and on the Template for Intervention Description and Replication.^14^ The variables extracted from each of study were the author(s), year of study and country in which it was conducted, along with the journal title. Summary information from each paper was recorded as the aim, purpose and/or objective of the study, and the research design. Detail of the methods included the sampling technique, recruitment strategy and process and the participant characteristics (including age range, gender). The frameworks used (TDF and/or COM‐B) were recorded, together with any rationale given for its use, and the measures used to operationalise the framework components. The main outcome and how it was measured were recorded, and any rewards that were given to participants for taking part. If the study was qualitative, the type of data analysis, together with how rigour (eg second coding) / saturation was assessed. If the study was a quantitative intervention study, the rationale for the intervention was recorded, together with the materials and procedure for the intervention, including how it was delivered, where and by whom, and whether there was any intervention tailoring (personalisation), modifications during the study, and fidelity. Finally, the findings of the study were extracted, including the main (behavioural) outcome, as well as any secondary outcomes.

## 3 RESULTS

**FIGURE 2:**
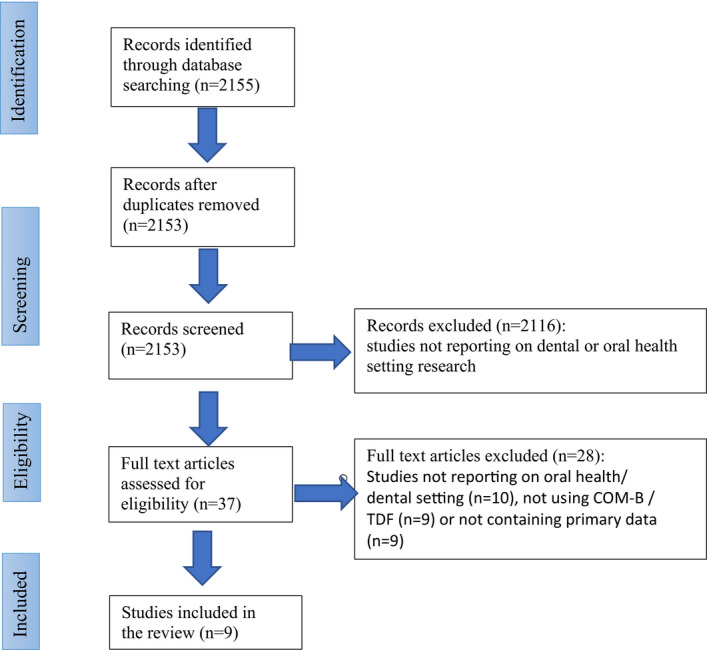
Study selection flow chart

A total of 2153 articles were screened and 2116 papers excluded because the papers did not report on research carried out in dental/oral health settings. The remaining 37 full papers were read independently by two authors (HB and KA) and were assessed for eligibility. In the end, 9 papers were included in the review. The process and flow diagram of study selection appear in Figure 2.

Table 3 lists the characteristics of the nine papers included in the review. The studies identified show that research was carried out internationally within Europe and North America. The published outlets for the research include both dentistry‐specific journals ^15^, ^16^, ^17^, ^18^, ^19^ (N = 5), Implementation Science journals (N = 3) and one in a Gerontology journal.^20^ A wide range of participants has been studied including: dentists,^21^, ^22^ dental teams ^23^ and parents of children.^16^ The studies used a variety of different designs, such as qualitative, natural experiments and surveys, although the definition of these designs was of variable description and quality. Most did not include an intervention as such, but several included natural experiments, for example exploring different behaviours before and after guidance was published.^18^, ^19^ In terms of the oral health‐related behaviours studied, researchers have examined a wide range of these, for example child tooth brushing, fluoride varnish application and non‐ or micro‐invasive management of proximal caries lesions. In some studies,^18^, ^21^ a multidisciplinary team including a behavioural scientist familiar with TDF and COM‐B was included in designing the research.

**TABLE 3:** Characteristics of included studies—Oral and dental research including the COM‐B/TDF

Authors/Year of Citation/Country	Aim/ Objective	Research design & framework employed	Key findings
Schwendicke et al (2018) New Zealand, USA, Germany	To identify barriers and enablers to dentists non‐ or micro‐invasively managing proximal caries lesions	Qualitative TDF (Partial)	*Barriers:* ‐ patients’ lacking adherence to oral hygiene instructions ‐ being high‐caries risk ‐ financial pressures and a lack of reimbursement ‐ unsupportive colleagues ‐ not undertaking professional development ‐ sense of anticipated regret *Enablers:* ‐ professional belief that early noncavitated lesions can be arrested ‐ having up‐to‐date info, supportive colleague/work environments ‐ working as part of a team ‐ having the necessary resources ‐ undertaking ongoing professional development ‐ membership of professional groups ‐ satisfaction from working in the patient's best interest.
Maramaldi et al (2018) USA	To propose empirically and conceptually supported interventions that might increase the capability and opportunity to provide oral hygiene care and oral cancer screening in long‐term nursing care facilities.	Qualitative Whole COM‐B (& Health Belief Model)	Findings suggest testing interventions targeting (a) high barriers/low opportunity/low service provision; (b) low capability/low service provision; and/or (c) high benefits/high capability/high service provision.
Jeggle et al (2019) Germany	To understand why German dentists are reluctant about selective carious tissue removal (SE), and to develop and test two interventions for changing dentists’ behaviour	Mixed methods TDF (Partial) + COM‐B Opportunity and Motivation +the Behaviour Change Technique Taxonomy Version 1	**Qualitative findings:** Barriers: ‐ lack of guidelines ‐ discrepancy between established and ‘new’ knowledge ‐ lack of routine Facilitators: ‐ understanding the biological foundations for SE ‐ having reliable criteria for determining the endpoint of SE **Intervention findings:** For both interventions, the outcome behaviour (simulated) improved significantly after the intervention (dentists were ‘less invasive’ after both interventions). There were no significant differences between the two interventions.
Marshman et al (2016) England	To explore parents’ experiences of tooth brushing with their young children and to establish barriers and facilitators to parental supervised brushing (PSB) at individual, interpersonal and environmental levels	Qualitative TDF (components not explicitly reported)	**Findings:** Parents: ‐not aware of national guidance on PSB ‐ had knowledge of tooth brushing practices ‐ intentions were to brush their children's teeth 2x day ‐Barriers to PSB were skills in managing children's behaviour and environmental influences on family life.
Gnich et al 2018 Scotland	To compare fluoride varnish application (FVA) pre‐ and post‐roll‐out of national financial incentive (and to explore the behavioural mechanisms underlying this) NB this study used the same participants as Gnich et al (2015)	Quantitative TDF (partial)	**Findings:** FVA rates increased over time for both groups; however, the novel incentive group had a greater increase than the continuous incentive group. Only 33% of GDPs reported ‘always’ varnishing increased risk 2‐5‐year‐olds'teeth following introduction of the financial incentive, 19% for standard risk children. Domain scores at time 2 increased more for novel incentive group for 5 domains: knowledge, social/professional role and identity, beliefs about consequences, social influences and emotion.
Gnich et al 2015 Scotland	To further understand what may influence fluoride varnish application (FVA) in GDP	Quantitative TDF (Partial)	**Findings:** Higher scores in 8 domains: Knowledge, Social/professional role and identity, Beliefs about consequences, Motivation and goals, Environmental context and resources, Social influences, Emotion and Behavioural regulation were associated with greater frequency of FVA. Four beliefs driving GDPs decision to apply FV:‐ ‐ FVA is a guideline recommended behaviour (Knowledge) ‐ FVA is perceived as an important part of the GDPs professional role (Professional role/identity) ‐ FV is something parents want for their children (Social influences) ‐FV is something GDPs really wanted to do (Emotion)
Amemori et al (2011) ‐ Finland	To improve our understanding of difficulties dental providers face in implementing TUPAC (Tobacco use Prevention and Cessation) guidelines and to provide an evidence‐based intervention design. Also to describe the development and use of a TDF Questionnaire	Quantitative TDF (Partial)	**Findings:** The internal consistency for the theoretical domains was in the acceptable range (0.5 ‐ 0.71). From 10 theoretical domains, three factors were extracted (motivation, capability and opportunity) that explain 70.8% of the variance. The domains environmental context and resources, beliefs about capabilities were identified as potential barriers to implementation.
Bonetti et al (2014) – Scotland UK	To examine the primary dental care management of patients on bisphosphonates before and after guidance publication, and to identify possible strategies to improve compliance via changing target beliefs	Quantitative TDF (Partial)	**Findings:** 10 months after the guidance was published (T2), all but one of the key behaviours were being performed more in line with guidance recommendations compared with reports of current practice in the survey conducted 2 months prior to publication (T1). At 22 months postpublication (T3), current practice had changed very little from T2. There was noncompliance for most behaviours still occurring at 10 months and at 22 months postpublication More positive attitude, greater perceived ability and greater motivation associated with significantly more guidance‐recommended management at every time point
Elouafkaoui et al (2015) – Scotland	To determine whether further intervention is required to translate the SDCEP (Scottish Dental Clinical Effectiveness Programme) guidance recommendations for the prevention and management of dental caries in children into practice.	Quantitative TDF (Partial)	**Findings:** The results highlight a gap between current practice and recommended practice. The majority of dentists do not ‘always’ perform recommended behaviours, and many are following treatment strategies specifically not recommended in the guidance. More positive attitude, greater capability and motivation were significantly associated with performing more guidance recommended risk assessment and prevention behaviours.

The majority of studies were based on the TDF only (n = 7), with 1 study employing both the COM‐B and Health Belief Model ^20^ and one using the TDF with COM‐B.^15^ All used a partial TDF, and the rationale for doing so was not always well reported or justified, although there was evidence for expert/consensus groups/panels advising on what aspects of the TDF to exclude. Some TDF domains were excluded on the authors’ judgement.^23^ For the qualitative studies included in the review, the type of analysis was not always reported or elaborated upon.^20^ However, details of saturation and rigour (eg second coding) were generally well‐reported. In terms of the operationalization of the TDF and COM‐B constructs, in the absence of a standardized measure, most authors developed their own TDF questionnaires. This development included using a qualitative approach in three studies, mixed methods in one study and five studies using quantitative surveys.

## 4 DISCUSSION

The COM‐B model has been widely used to identify what needs to change in order for interventions to be effective in changing health‐related behaviour,^24^ with the TDF also being increasingly utilized. The aim of our review was to identify oral health research that had adopted the COM‐B and/or TDF as a guide to the development of the study.

It was encouraging to see that some researchers are adopting these psychological frameworks to design studies to further our understanding of correlates of oral health‐related behaviour and that they are doing so by including behavioural scientists with expertise in these domains, in the study team. There are several common limitations with the studies published to date. However, it would appear that at present the use of the COM‐B and TDF is still somewhat limited with only a small number of studies explicitly using either framework. Interestingly, of the nine studies included in the review, all but one of them included dental professionals and/or health staff as participants. This was because the studies to date have primarily focused on understanding the behaviours underpinning clinical practice around dental guidelines and practices, rather than on individual oral health behaviours per se. Additionally, most of those studies that have utilized either COM‐B or TDF have not employed them in their entirety. This may have arisen from practical constraints. In future, we recommend that they are used in their entirety, where this is not possible the choice of components should be informed by key stakeholders or, in the case of intervention development, by initial qualitative interviews with participants.

Where all components are not included, it means that researchers may not classify psychological and behavioural constructs in a consistent and transferable manner across studies. This finding is in line with previous attempts to provide a synthesis of published studies of oral health‐related behaviour change, showing that often components of frameworks rather than frameworks in their entirety seem to be utilized in oral health interventions.^25^, ^26^, ^27^, ^28^ In future, it could be that the observed heterogeneity in oral health interventions could be addressed by using the COM‐B and TDF to classify the elements of each intervention to allow for a greater and more consistent synthesis of the findings.

From a practical point of view, while it is encouraging to see that the TDF has been used in a small number of oral and dental studies, the COM‐B model is employed rarely. This is a surprising find given its central role in designing behavioural interventions outside of oral health. One reason may by that COM‐B concepts still appear difficult to comprehend and hence impractical to adopt. For example, with the TDF, it is known that healthcare professionals have struggled in the past to understand how it can help their research efforts,^10^ which led to efforts to explicitly offer researchers clarification on the use of the framework.^29^ For COM‐B, there has recently been an attempt to offer researchers clarity in operationalising its components by using a standardized questionnaire to assess each component which may increase the model's use.^30^

In terms of recommendations for future research, in which self‐report tools are used, we would advise that for the COM‐B, researchers use the recently published brief, validated measure for quantitative work ^20^ and adapt it for their study, rather than designing their own tools. For the TDF, we propose researchers consider using the tool in its entirety or that they clearly outline the rationale and process for including only some domains. Such self‐reported tools could be used alongside proxy clinical measures (eg plaque) or behavioural observations (eg tooth brushing, clinical practice) where possible. For qualitative studies, we would encourage researchers to outline the type of analysis employed with a clear rationale for their choice of domains. Finally, multidisciplinary teams including behavioural scientists such as health psychologists should ensure the use of these frameworks is supported by relevant expertise.

It should also be highlighted that COM‐B is not without its criticisms. For example, Ogden ^31^ acknowledges that there is much to be lauded in the goals of COM‐B, such as the aim of promoting evidence‐based practice and better reporting of interventions. However, she argues that the aim of specifying which intervention tools should be used for a particular behaviour ignores the need for flexibility, variability and change according to how that individual feels thinks, looks, behaves or responds at any given time. She argues we should be celebrating this variability rather than removing it. Thus, researchers (and practitioners) should be aware of these potential limitations when applying and evaluating the COM‐B.

It is important to highlight the limitations of this review. Firstly, despite the fact that we used SCOPUS to search for eligible studies, it is possible that we failed to identify some relevant studies. However, to mitigate this, we searched for relevant studies using reference lists of all included studies. Secondly, our review may have introduced bias through inclusion of only those peer reviewed studies published in journals. Thus, we may not have included some of the most up‐to‐date research on the COM‐B and TDF. However, the focus of our review was to specifically assess the published work on this model and framework.

In conclusion, this review has identified that some oral health researchers are adopting psychological frameworks such as COM‐B and TDF to ground their research. However, this is currently limited both in the small number of studies adopting these frameworks and the comprehensiveness of their adoption. We recommend that researchers interested in the development of approaches to understanding the psychological determinants of oral health‐related behaviour, and in the design of interventions to enhance such behaviour, adopt the COM‐B and TDF framework and associated elements in such research as well as use standardized methodologies to the design and conduct of interventional research.^5^

## CONFLICT OF INTEREST

The authors have no conflicts of interest to declare.

## AUTHOR CONTRIBUTIONS

SB commented on the manuscript; JTN carried out initial searches and commented on the manuscript; HB and KA carried out searches, screened papers, drafted and revised the manuscript.
